# An Efficient Ensemble Learning Method for Gene Microarray Classification

**DOI:** 10.1155/2013/478410

**Published:** 2013-08-14

**Authors:** Alireza Osareh, Bita Shadgar

**Affiliations:** Department of Computer Engineering, Islamic Azad University, Dezful Branch, Dezful 313, Iran

## Abstract

The gene microarray analysis and classification have demonstrated an effective way for the effective diagnosis of diseases and cancers. However, it has been also revealed that the basic classification techniques have intrinsic drawbacks in achieving accurate gene classification and cancer diagnosis. On the other hand, classifier ensembles have received increasing attention in various applications. Here, we address the gene classification issue using RotBoost ensemble methodology. This method is a combination of Rotation Forest and AdaBoost techniques which in turn preserve both desirable features of an ensemble architecture, that is, accuracy and diversity. To select a concise subset of informative genes, 5 different feature selection algorithms are considered. To assess the efficiency of the RotBoost, other nonensemble/ensemble techniques including Decision Trees, Support Vector Machines, Rotation Forest, AdaBoost, and Bagging are also deployed. Experimental results have revealed that the combination of the fast correlation-based feature selection method with ICA-based RotBoost ensemble is highly effective for gene classification. In fact, the proposed method can create ensemble classifiers which outperform not only the classifiers produced by the conventional machine learning but also the classifiers generated by two widely used conventional ensemble learning methods, that is, Bagging and AdaBoost.

## 1. Introduction

Previous studies have shown that gene microarray data analysis is a powerful and revolutionary tool for biological and medical researches by allowing the simultaneous monitoring of the expression levels of tens of thousands of genes [1]. 

This is done by measuring the signal intensity of fluorescing molecules attached to DNA species that are bound to complementary strands of DNA localized to the surface of the microarray. Usually a ratio of intensities is calculated for each probe or gene, corresponding to two different labeled populations of reverse-transcribed mRNA.

Having captured the spot intensities, the obtained intensities undergo a normalization preprocessing stage to remove systematic errors within the data [2]. Early application of microarrays to the study of human disease conditions rapidly revealed their potential as a medical diagnostic tool [3, 4]. This is a class prediction problem to which supervised learning techniques are ideally suited. 

Some studies have been reported on the application of microarray gene expression data analysis for molecular cancer classification [5, 6]. In fact, Microarray analysis has demonstrated that accurate cancer diagnosis can be achieved by performing microarray data classification, that is, by constructing classifiers to compare the gene expression profile of a tissue of unknown cancer status to a database-stored expression profile from tissues of known cancer status. 

Usually, microarray classification process comprised of two successive steps, that is, feature selection and classification. So far, many machine learning algorithms have been introduced, and many of them have been employed for both steps, including the techniques of feature selection [7], classification techniques, for example, K-NN [8], support vector machines [9, 10], and neural networks [11]. Most of the existing research works attempt to choose an optimal subset of genes and then generalize an accurate classification model based on the selected genes. 

The microarray data measures the expressions of tens of thousands of genes, producing a feature vector that is high in dimensionality and that contains much irrelevant information. This dimensionality degrades classification performance. Moreover, datasets typically contain few samples for training (e.g., lung dataset [12] contains 12535 genes and only 181 samples), leading to the curse of dimensionality problem. It is essential, therefore, to find efficient methods for reducing the size of the feature set.

To avoid the curse of dimensionality problem, gene selection plays a crucial role in DNA microarray analysis. Another important reason to reduce dimensionality is to help biologists to identify the underlying mechanism that relates gene expression to diseases.

Indeed, the microarray data is associated with various uncertainties such as microarray data, gathering process which include fabrication, hybridization and image processing. These uncertainties always add various sources of noise [13]. Because of the impact of different uncertainties together with the lack of labeled training samples, the conventional machine learning techniques face complicated challenges to develop reliable classification models. Quite often selecting only a few genes can discriminate a majority of training instances correctly [14]. However, the generalization ability of such classifier model based on a few principal genes and a limited number of labeled training instances cannot be guaranteed. 

It is therefore essential to develop general approaches and robust methods that are able to overcome the limitation of the small number of training instances and reduce the influence of uncertainties so as to produce reliable classification results. The motivation for this study is to utilize robust ensemble methods that are less sensitive to the selection of genes and are capable of removing the uncertainties of gene expression data.

Ensemble methodology is an efficient technique that has increasingly been adopted to combine multiple learning algorithms to improve overall prediction accuracy [15]. These ensemble techniques have the advantage to alleviate the small sample size problem by averaging and incorporating over multiple classification models to reduce the potential for overfitting the training data*  *[16]. In this way the training data set may be used in a more efficient way, which is critical to many bioinformatics applications with small sample size.

Much research has shown the promise of ensemble learning for improving the accuracy in classifying data under uncertainties [15, 17]. However, a necessary and sufficient condition for an ensemble to outperform its individual members is that the base classifiers should be accurate and diverse [18]. An accurate classifier is one that has an error rate of better than randomly guessing classes for new unseen samples. On the other hand, two classifiers are said to be diverse if their decisions are different when classifying the same new instance, that is, if the individual classifiers do not always agree.

The most popular ensemble methods utilize a base classification algorithm to differently permutated training sets. Examples of these techniques include AdaBoost, Bagging, Random Subspace, Random Forest, and Rotation Forest [19]. AdaBoost has become a very popular choice for its simplicity and adaptability [20]. This algorithm builds an ensemble of classifiers by utilizing a specified base learning algorithm to successive obtained training sets that are formed by either resampling from the original training set or reweighting the original training set according to a set of weights maintained over the training set [20]. Thus, AdaBoost attempts to produce new “strong” classifiers that are able to better predict the hard instances for the previous ensemble “weak” members. 

In Bagging, each base classifier is constructed on a bootstrap sample of the original training data, that is, a random sample of instances drawn with replacement and having the same size as the original training data. Ensemble classification is achieved by means of majority voting, where an unlabeled unseen data is assigned the class with the highest number of votes among the individual classifiers' predictions [21]. 

A successful variation upon Bagging is the Rotation Forest. Rotation Forest is an ensemble classification approach which is built with a set of decision trees. For each tree, the bootstrap samples extracted from the original training set are adopted to construct a new training set. Then the feature set of the new training set is randomly split into some subsets, which are transformed individually. Since a small rotation of axes may build a complete different tree, the diversity of the ensemble system can be guaranteed by the transformation [22].

Compared with the other proposed ensemble approaches, such as AdaBoost [23], Bagging [24], and Random Forest [25], Rotation Forest is more robust because it can always enhance the generalization ability of the individual classifiers and the diversity in the ensemble at the same time. 

C. Zhang and J. Zhang [19] proposed a novel ensemble classifier generation method RotBoost through combining Rotation Forest and AdaBoost. In this new ensemble method, the base classifier in Rotation Forest algorithm is replaced with AdaBoost. The experimental results show that RotBoost performs better than either Rotation Forest or AdaBoost when using some non-microarray gene-related data sets from the UCI repository.

Here, we inspired from RotBoost technique and suggest a two-stage ensemble learning methodology by integrating fast correlation-based filter feature selection and independent component analysis- (ICA-) based RotBoost ensemble classification. Indeed, to verify the efficiency of the proposed method on gene-related data, 8 publically available gene microarray benchmark datasets are analyzed. 

To this end, we accomplish a comparative study of RotBoost efficiency against several other ensemble and single classifier systems including AdaBoost, Bagging, Rotation Forest single tree, and support vector machines (SVMs). Moreover, to achieve the optimum arrangement and parameters, different variations of RotBoost such as PCA-based and ICA-based are compared, following an implementation of several feature selection approaches.

In terms of generalization accuracy, ICA-based RotBoost ensemble in conjunction with fast correlation-based filter demonstrated superior average performance over all considered ensemble classifiers and is therefore recommended as an efficient classification technique for the prediction of new gene microarray class labels.

The rest of this paper is organized as follows. In Section 2, the framework of RotBoost is described in detail. In Section 3, the experimental results and corresponding discussions are presented.  Section 4 concludes the paper. 

## 2. Materials and Methods

### 2.1. The Description of RotBoost Ensemble Classification

As it was stated before, RotBoost is constructed by integrating the ideas of Rotation Forest and AdaBoost ensemble classifier generation techniques with the aim of achieving even lower prediction error than either of these individual techniques.

Rotation Forest is an ensemble method which trains *L* decision trees independently, using a different set of extracted features for each tree [25]. Let *x* = [*x*_1_,…, *x*_*n*_]^*T*^ be an example described by *n* features (attributes) and let  *X* be an *N* × *n* matrix containing the training examples. Assuming that *w* is the set of class labels {*w*_1_,… , *w*_*m*_}, from which *Y* takes values. Training a base classifier *C*_*i*_ involves using the training data to formalize a mapping of the input variable space onto the binary response variable, *Y*. If the feature set *F* is split randomly into *K* subsets with approximate size, there will be the ensemble of  *L*  classifiers denoted by *D* = {*D*_1_,…, *D*_*L*_}. To construct the training set for an individual classifier *C*_*i*_, we split *F* into *K* disjoint subsets randomly where each feature subset contains *M* = *n*/*K* features [23]. Let *F*_*ij*_ be the *j*th subset of features for training classifier  *C*_*i*_ and *X*_*ij*_ the dataset *X* for the features in *F*_*ij*_. Now, for each subset, we select a nonempty subset of classes from*X*_*ij*_ randomly. Then a bootstrap subset of input instances is drawn to form a new training set, which is denoted by *X*_*ij*_′. Subsequently, a transformation such as PCA is applied on  *X*_*ij*_′ to generate the coefficients in a matrix *Q*_*ij*_, denoted by the coefficients *a*_*ij*_^(1)^,…, *a*_*ij*_^(*Mj*)^. Therefore, the size of each *X*_*ij*_′ is *M* × 1.

Finally, we canconstruct a sparse rotation matrix *R*_*i*_ with the obtained coefficients in matrix *Q*_*ij*_, as follows [22]:

(1) $$\begin{array}{c} R_{i}=\left[\begin{array}{ccc} a_{i1}^{\left(1\right)},\ldots ,a_{i1}^{\left(M_{1}\right)} & \left\{0\right\}\ldots & \left\{0\right\}\\ \left\{0\right\} & a_{i2}^{\left(1\right)},\ldots ,a_{i2}^{\left(M_{2}\right)} & \left\{0\right\}\\ \left\{0\right\} & \left\{0\right\} & a_{iK}^{\left(1\right)},\ldots ,a_{iK}^{\left(M_{K}\right)} \end{array}\right]. \end{array}$$

The columns of *R*_*i*_ are rearranged according to the original feature sequence, and the rearranged rotation matrix is indicated by *R* ^*a*^_*i*_. The new obtained transformed training set for classifier  *C*_*i*_ is shown by *XR* ^*a*^_*i*_. During the test stage, given a test input instance *x*, let *d*_*ij*_(*xR* ^*a*^_*i*_) be the probability produced by the classifier *C*_*i*_ to the hypothesis that *x* belongs to class *w*_*j*_. Then the confidence for a class can be obtained as follows:

(2) φj(x)=1L∑i=1Ldij(xRai), j=1,…,m.

Now, an unknown input sample *x* is assigned to the class with the largest confidence. In RotBoost, the base classifiers *C*_*i*_ in Rotation Forest are replaced by AdaBoost classifiers. RotBoost offers a potential computational advantage over AdaBoost in that it has the ability to execute in parallel. In fact, each subensemble classifier formed by AdaBoost can be learned independently of the other ones.  Pseudocode 1 illustrates this algorithm.

**Figure:**
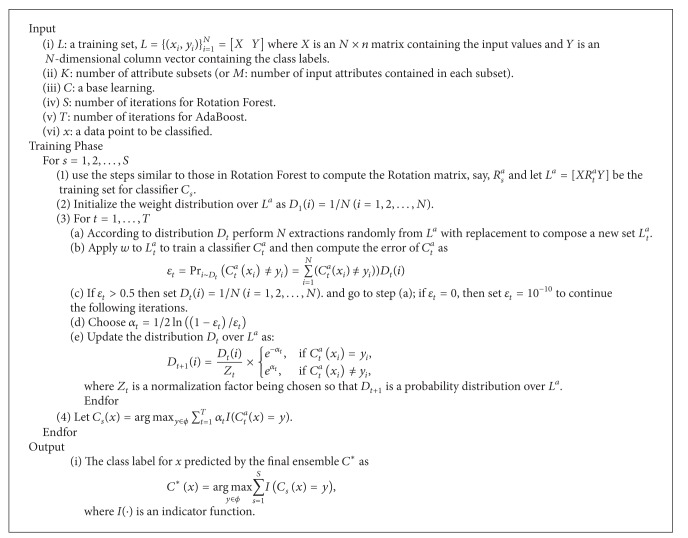
The RotBoost pseudocode.

When employing the RotBoost classification algorithm, some parameters are required to be defined beforehand. The values of the parameters *S* and *T* that, respectively, specify the numbers of iterations done for Rotation Forest and AdaBoost should be subjectively fine-tuned, and the value of *K*(or *M* which represents the number attributes in each subspace) can be selected to be a moderate value according to the size of the feature set *F*. Indeed, the decision trees are utilized as the individual base classifiers of the final constructed RotBoost ensemble predictor. 

### 2.2. Transformation Methods

As it was already mentioned, the purpose of rotation-based ensemble classifiers such as Rotation Forest and RotBoost is to increase the individual classifier performance and the diversity within the ensemble. Thus, a full feature set is obtained with all the transformed features for each considered tree in the ensemble. Because a small rotation of axes may construct a complete different tree, the diversity of the ensemble classifier can be guaranteed by the selected transformation.

There are largely two kinds of transformation methods, that is, PCA and ICA. PCA projects the data into a new space spanned by the principal components [26]. In contrast to PCA, ICA decomposes an input dataset into components so that each component is statistically as independent from the others as possible. It appears that ICA has a greater advantage over PCA in many aspects. First, it provides a better probabilistic model of the data, which can better identify where the data concentrate in *n*-dimensional space. Second, it can find a not necessarily orthogonal basis, which may reconstruct the data better than PCA in the presence of noise. Finally, it is sensitive to high-order statistics in the data, not just the covariance matrix [27]. Here, for the sake of comparison, we experiment with both PCA and ICA transformation methods and will report on their efficiency for our gene microarray classification task later on. 

### 2.3. Gene Selection

Available training data sets for classification of cancer types generally have a fairly small sample size compared to the number of genes involved. This fact poses a challenging difficulty to some classification methodologies due to training data limitations. To be more specific, in a typical microarray dataset, there are thousands of gene features. Then if RotBoost ensemble classifier is applied to classify such dataset directly, a rotation matrix with thousands of dimensions is required for each tree, which greatly increases the computational complexity. 

On the other hand, as only a small subset of genes is potentially relevant for distinguishing the sample classes, feature selection technique is crucial to reduce the number of features, removes irrelevant, noisy, and redundant data, and results in acceptable classification accuracy.

There are two broad categories for feature selection algorithms, filter model or wrapper [28]. The filter model relies on general characteristics of the training data to choose best features without involving any learning algorithm. 

The wrapper models, on the contrary, depends on feature addition or deletion to compose subset features and uses an evaluation function with a predetermined learning algorithm to estimate the subset features. Although these models tend to find features better suited to the learning algorithm resulting in superior learning performance, they also tend to be more computationally expensive than the filter model [29]. 

When the number of features becomes very large, the filter model is usually chosen due to its computational efficiency. Here, we utilize fast correlation-based filter (FCBF) as previous experiments [30] suggest that FCBF is an efficient and fast feature selection algorithm for classification of high dimensional data. FCBF model uses interdependence of features together with the dependence to the class and can achieve high degree of dimensionality reduction. This in turn improves classification accuracy with predominant selected features.

FCBF begins by selecting a subset of relevant features whose *C*-correlations are larger than a given threshold and then sorts the relevant features in descending order in terms of *C*-correlation. Using the sorted feature list, redundant features are eliminated one by one in a descending order. The remaining feature subset thus contains the predominant features with zero redundant features in terms of *C*-correlation [31].

### 2.4. Datasets

Here, we utilized 8 publicly available benchmark datasets [32]. A brief overview of these datasets is summarized in Table 1. Preprocessing is an important step for handling gene expression data. This includes two steps: filling missing values and normalization. For both training and test dataset, missing values are filled using the average value of that gene. Normalization is performed so that every gene expression has mean equal to 0 and variance equal to 1. In summary, the 8 datasets had between 2 and 5 distinct diagnostic categories, 60–253 instances, and 2000–24481 genes. 

## 3. Results and Discussion

In our experiments, we choose four representative feature selection algorithms, that is, ReliefF, correlation-based filter selection (CFS), minimum redundancy maximum relevance (mRMR), and general signal to noise ratio (GSNR) in comparison with FCBF. 

ReliefF [33] is an extension of the original Relief algorithm [34] that adds the ability of dealing with multiclass problems, and it is more robust and capable of dealing with incomplete and noisy data. The Relief family methods are especially attractive because they may be applied in all situations, have low bias, include interaction among features, and may capture local dependencies that other methods miss [35].

The CFS method is based on test theory concepts and relies on a set of heuristics to assess the adequacy of subsets of features. These heuristics take into account both the usefulness of individual features to predict the class label as well as their correlation [34].

 The mRMR criterion computes both the redundancy between features and the relevance of each feature. Redundancy is computed by the mutual information (MI) between pairs of features whereas relevance is measured by the MI between each feature and the class labels. The mRMR method has also been applied successfully to microarray data [36].

 The GSNR is a measure of the ratio between intergroup and intragroup variations. Higher GSNR values indicate higher discrimination power for the gene. GSNR selects *m* genes in the descent order, and the best subset of genes is selected based on a predefined description. 

 In order to reduce the computational complexity of the problem at hand and select the most informative genes, we run all 5 feature selection algorithms against each dataset and obtain the number of selected features for each algorithm.  Table 2 shows the number of genes which are selected by these feature selection algorithms for each individual microarray gene dataset. As it can be seen, the number of selected genes for each processed gene dataset is different and depends on the choice of a feature selection algorithm.

It should be noted that both mRMR and GSNR algorithms provide an ordered list of the initial genes (features) according to the genes importance and discrimination power. Here, for the sake of comparison, we experimentally retained the top 10% of the sorted genes by each of these two feature selection algorithms. This in turn leads to less computational cost in experiments. 

From Table 2, it is obvious that FCBF achieves the highest level of dimensionality reduction by selecting the least number of discriminative genes. This is consistent with the theoretical analysis about FCBF's ability to identify and ignore redundant features.

To evaluate the gene classification accuracy of selected top genes by each feature selection algorithm, a single decision tree learning algorithm is utilized. The learning algorithm is applied to the original gene datasets as well as each newly obtained dataset containing only the selected genes, and in each case the final overall accuracy is measured. 

**Figure 1:**
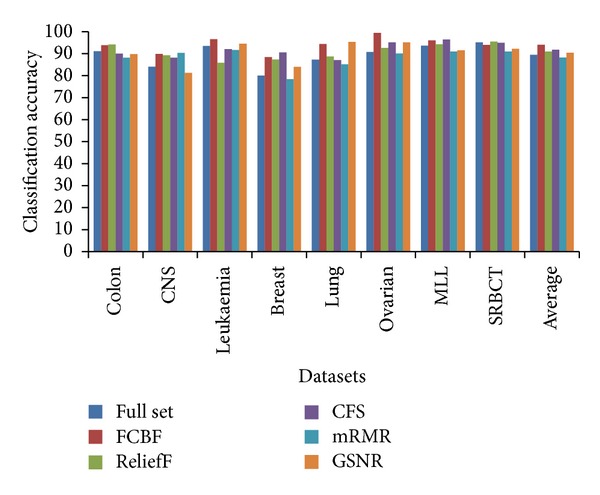
Classification accuracy of decision tree classifier on selected genes of 8 datasets based on different feature selection algorithms.

Figure 1 summarizes the learning accuracy of decision tree classifier on different feature sets. Considering the averaged accuracy over all data sets, we observe that, in general, FCBF improves the accuracy of decision tree classifier; it also outperforms the other four feature selection algorithms. Indeed, from individual accuracy values, we observe that for all the datasets except SRBCT, FCBF can highly increase the overall gene classification accuracy. On the other hand, CFS method achieves the second best classification accuracy, and both relief and GSNR accomplish more than 90% average accuracy. 

However, as it was already mentioned, a single classifier such as decision tree is far from an accurate classifier when applied to the problem of gene microarray classification which usually confronts several challenges such as curse of dimensionality, small sample size datasets, and huge amount of noise and uncertainties. 

To cope with these challenges and to develop a more robust and accurate learning method, ensemble learning methodology is utilized. The datasets are first preprocessed, and then to reduce the computational complexity and select the most informative genes, FCBF is applied to these datasets. Having chosen the best discriminative features, the ensemble classifiers including RotBoost, Rotation Forest, AdaBoost, and Bagging are developed and learned using these features. In all ensemble experiments a classification tree [16] was exploited as the base learner because it is sensitive to the changes in its training data.

In order to provide a fair comparison, for all utilized ensemble techniques 100 trees are trained to constitute the corresponding ensemble classifiers. With respect to RotBoost, the number of iterations for Rotation Forest and AdaBoost both fine-tuned to be *S* = *T* = 10 (to properly balance the trade-off between these two algorithms). 

 In theory the parameter *M* should be selected to be a moderate value according to the size of the feature set *F*. Therefore, it seems that the performances of RotBoost would be changed with different number of features contained in each feature subset (*M*). But when comparing the results obtained by setting *M* ranged between 1 and 20, it is found that the overall gene classification results under different conditions vary slightly and none of the values take obvious advantage. So there was no consistent relationship between the classification accuracy and *M*, which was also pointed out in [37]. Here, the option*M* = 3 was the optimum choice to establish a proper balance between the overall classification accuracy and the diversity of the based learners for most examined gene datasets. 

To find the most promising transformation method and preserve both diversity and accuracy of the base classifiers, we conduct experiments with two well-known transforms, that is, PCA and ICA. When the initial genes are transformed by either PCA or ICA, all of the principal components or independent components are kept to preserve the discriminatory information. Following transformation, the axes are rotated optimally. Despite the conventional approach of choosing some directions for good discriminate capability, the rotation mainly contributes to the generation of diversity among the classifiers without weakening the individual classifiers. Thus, an acceptable trade-off between diversity and accuracy can be maintained simultaneously.

In many earlier works, researchers typically split the original dataset into two parts, that is, a training set and a test set in a random fashion. Gene selection is then performed on the training set, and the goodness of selected genes is assessed from the unseen test set [31]. 

However, due to the small number of instances in gene microarray datasets, such an approach can lead to unreliable results. Instead, Ambroise and McLachlan [38] suggested splitting the data using 10-fold cross-validation or 0.632 + bootstrap. A comparative study of several different error estimation techniques on microarray classification shows that 0.632 + bootstrap can be more appropriate than other estimators including resubstitution estimator, cross-validation, and leave-one-out estimation [39].

Therefore, in this work, we deployed a balanced 0.632+bootstrap technique to evaluate the performance of the gene selection algorithm considered in this study. The 0.632 + bootstrap requires sampling a training set with a replacement manner from the original dataset. The test set is then made by those samples excluded from the training dataset. Finally, the 0.632 + bootstrap is repeated *n* times, and the final bootstrap error is estimated as follows:

(3) $$\begin{array}{c} E=\frac{1}{n}\sum_{i=1}^{n}\left(0.368\alpha_{i}+0.632\beta_{i}\right), \end{array}$$

where *α*_*i*_ and *β*_*i*_ are the training error and test error on the *i*th*  *resampling stage. Following the work in [14], here, the bootstrap samples are experimentally formed with *n* = 15 replicates. It is worth to note that the feature selection is then carried out using only the training samples. Finally, the test error (classification accuracy) is estimated on the unseen test samples using (3).

Table 3 presents the RotBoost mean classification accuracy against the considered 8 gene datasets when transformation matrix is chosen to be either PCA or ICA where the values following “±” denote the related standard deviations. In order to explore whether RotBoost is significantly better or worse than other ensemble/nonensemble methods statistically, a one-tailed paired *t*-test is considered with significance level *α* = 0.05  and the results for which a significant difference with RotBoost was found are marked with a bullet or an open circle next to them. A bullet next to a result indicates that RotBoost is significantly better than the corresponding method. An open circle next to a result denotes that RotBoost performs significantly worse than the corresponding method. In the triplet labeled “Win-Tie-Loss” in the last row of Table 3, the first value denotes the number of gene datasets on which RotBoost operates considerably better than the corresponding algorithm; the second value stands for the number of datasets on which the difference between the performance of RotBoost and that of the corresponding algorithm is not significant; the third one indicates the number of datasets on which RotBoost performs significantly worse than the compared algorithm.

As it can be noted from Table 3, in 5 cases out of 8, ICA-based RotBoost learners could outperform their PCA-based counterparts in terms of higher classifications accuracies and lower standard deviations. On the other hand, in 2 cases, that is, Leukaemia and SRBCT datasets, the results obtained by both methods are not significantly different, and in 1 case (Ovarian dataset) the PCA-based RotBoost classifier could surpass the ICA-based learner. It is also necessary to point out that all these experiments have been accomplished on the best discriminative genes already selected by FCBF algorithm.

It is well known that no algorithm can hold a general advantage in terms of generalization capability over another one across all possible classification tasks. However, the relative advantage of an algorithm is possible across a set of real-world tasks. Considering that the 8 gene microarray datasets include different characteristics in terms of number of samples, genes, classes and the type of the cancer to which these data is related to; overall, ICA-based RotBoost classifier seems to be more effective than PCA-based RotBoost.

Following our decision on the choice of the transformation method, we accomplish a comparative study of ICA-based RotBoost accuracy performance against other ensemble and an independent single classifier system including AdaBoost, Bagging, Rotation Forest, single tree, and SVMs. 

Specifying a SVMs classifier requires two parameters, that is, the kernel function and the regularization parameter *C*. In this study, the SVMs classifiers are evaluated based on Gaussian radial bases functions (RBF). In order to obtain the optimal value for the SVMs regularization parameter *C* and the parameter of kernel functions (*σ*), we experiment with different SVMs classifiers using a 5-fold cross-validation technique. The performance of the optimum selected SVMs is measured in terms of classification accuracy (Table 4).

Table 4 summarizes the mean classification accuracy of each classification method on the considered datasets. As can be seen from this table, RotBoost methodology performs significantly better than Single Tree, SVMs and Bagging, algorithms. When compared with Rotation Forest, the statistically significant difference is favorable in 6 datasets, although the Rotation Forest could surpass the RotBoost when working on Breast and Ovarian datasets. Indeed, RotBoost is seen to outperform AdaBoost in most cases even though the advantage of RotBoost is not significant in 1 dataset and tie is occurred on the remaining 2 datasets. 

An important issue in ensemble learning methodology is the ability to establish a proper balance between the diversity and the accuracy of the constituted base learners. That is, the base learners utilized in a robust ensemble classifier should be of high classification accuracy and avoid making coincident misclassification errors which in turn necessitate the diverse learners. Thus, a sample misclassified by a base learner will be corrected by others, so the fused outputs are more accurate than that of the best individual classifier [37].

On the other hand, the diversity usually conflicts with the accuracy of base learners; that is, the more accurate the base learners are, the lower the diversity among them is. In practice, it appeared to be difficult to define a single measure of diversity and even more difficult to relate that measure to the ensemble performance in a neat and expressive dependency. Here, to investigate the ability of the proposed ICA-based RotBoost ensemble to build accurate and diverse base learners efficiently, the pairwise diversity measure is utilized [40]. 

This diversity measure evaluates the level of agreement between a pair of base learners while correcting for chance, which is named as Kappa statistic. For *c* class labels, *k* is defined on the *c* × *c* coincidence matrix **M** of the two classifiers. The entry *m*_*k*,*s*_ of **M** is the proportion of the data set, which *D*_*i*_ labels as *w*_*k*_ and *D*_*j*_ labels as *w*_*s*_. Now, the agreement between *D*_*i*_ and *D*_*j*_ is given by

(4) $$\begin{array}{c} k_{i,j}=\frac{\sum_{k}m_{k,k}-ABC}{1-ABC}, \end{array}$$

where  ∑_*k*_ *m*_*k*,*k*_ is the observed agreement between the classifiers, and “*ABC*” which is named agreement-by-chance is defined as follows [41]:

(5) $$\begin{array}{c} ABC=\sum_{k}\left(\sum_{s}m_{k,s}\right)\left(\sum_{s}m_{s,k}\right). \end{array}$$

If the classifiers decisions are the same, *k* = 1 and *k* = 0 represent the case when the classifiers are entirely independent, and the agreement of the two classifiers equals that expected by chance [41]. In theory, there are (*P* − 1) × *P*/2 pairs of classifiers *D*_*a*_ and *D*_*b*_ for an ensemble with *P* base learners. For a typical kappa-error diagram, *x*-axis is the *k* for the pair, and *y*-axis is the averaged individual error, which is calculated by *E*_*a*,*b*_ = (*E*_*a*_ + *E*_*b*_)/2, where *E*_*a*_ and *E*_*b*_ refer to the error rates of pairs of classifiers *D*_*a*_ and *D*_*b*_, respectively. It is worth to note that a small value of *k* denotes superior diversity among the learners, and in turn a small value of *E*_*a*,*b*_ presents less error rate between the classifiers.

Table 5 summarizes the kappa-error values for typical Lung cancer dataset with FCBF gene selection method in terms of the centroids of different ensembles. From this table it is clear that the ICA-based RotBoost provides the highest pairwise accuracy, and the second best accuracy is achieved by PCA-based RotBoost. On the contrary, AdaBoost presents the best diversity at the cost of relatively poor classification accuracy. Bagging also presents the second best diversity but low classification accuracy. 

**Figure 2:**
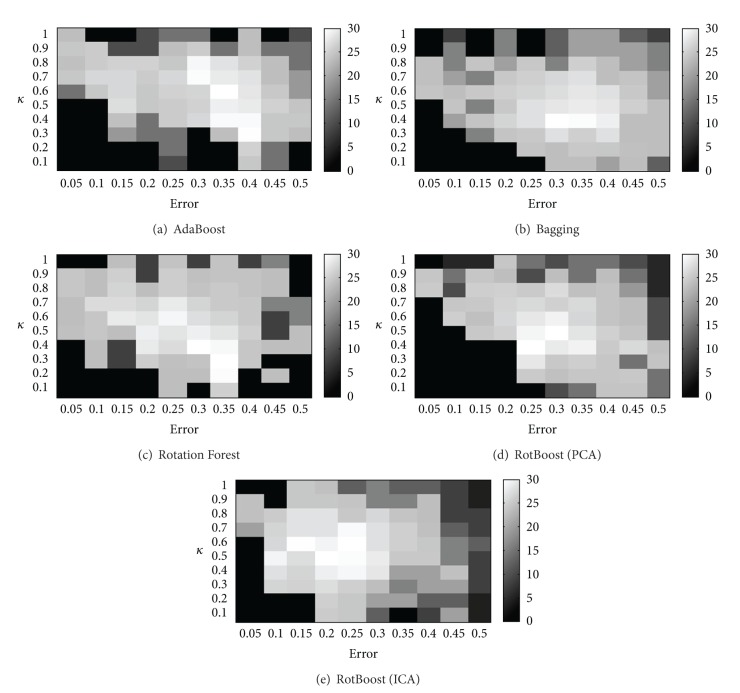
Kappa error diagrams for the Lung dataset using different ensemble algorithms.

 When outlining the kappa-error diagrams, the diagrams of different ensembles are greatly overlapping, and the distances between the centroids are small. Therefore, a three dimensional histogram with a density underneath plot is usually utilized to clearly demonstrate the distribution of the results achieved by each ensemble method.  Figure 2 illustrates these histograms against the typical Lung cancer dataset. 

We perform these experiments on all 8 gene datasets, and overall the ICA and PCA RotBoost methods perform best in terms of accuracy with average classification accuracy about 98.0% and 96.3%, respectively. Furthermore, these ICA and PCA RotBoost ensemble methods achieve average kappa values of 0.48 and 0.55, respectively. This is coincident with the observation previously given that the diversity usually conflicts with the accuracy of the base learners. However our experiments indicate that the ICA-based RotBoost ensemble could establish a proper balance between the diversity and the accuracy of the constituted base learners.

## 4. Conclusions

 In this work, we addressed RotBoost ensemble classification method to cope with gene microarray classification problems. This ensemble classifier method is a combination of Rotation Forest and AdaBoost techniques which in turn preserve both desirable features of an ensemble architecture, that is, accuracy and diversity. To overcome the limitation of the small number of gene instances and curse of dimensionality and in order to select a small subset of most informative genes, 5 representative feature selection algorithms, that is, ReliefF, CFS, mRMR, GSNR, and FCBF were applied. Then, RotBoost was employed on the selected genes. Here, we experimented with 2 different transformation matrixes, that is, PCA and ICA towards RotBoost implementation.

 To assess the efficiency of RotBoost algorithm different ensemble/nonensemble techniques including Rotation Forest, AdaBoost, Bagging single tree, and SVMs were also deployed. The experimental results revealed that the combination of the FCBF feature selection and ICA-based RotBoost ensemble with several base learners is a robust method for microarray classification. The proposed method achieved the highest averaged generalization ability compared to its counterparts and denoted an acceptable level of diversity among the learners for majority of the analyzed benchmark datasets.

## Figures and Tables

**Table 1:** Summary of benchmark gene microarray datasets.

Dataset	# Total genes (*T*)	# Instances (*n*)	# Classes (*C*)
Colon tumor	2000	62	2
Central nervous system	7129	60	2
Leukaemia	6817	72	2
Breast cancer	24481	97	2
Ovarian cancer	15154	253	2
MLL	12582	72	3
SRBCT	2308	83	4
Lung cancer	12533	181	5

**Table 2:** Number of selected genes for each gene selection algorithm.

Dataset	Initial gene numbers	FCBF	ReliefF	CFS	mRMR	GSNR
Colon	2000	14	25	31	100	100
CNS	7129	28	28	40	356	356
Leukaemia	7129	51	104	50	356	356
Breast	24481	90	131	130	1224	1224
Lung	12553	100	432	299	628	628
Ovarian	15154	30	120	103	587	587
MLL	12582	97	295	327	629	629
SRBCT	2308	82	97	82	115	115

**Table 3:** Classification results obtained by RotBoost ensemble learning against typical 8 gene datasets in terms of PCA/ICA transformation methods.

Dataset	ICA_based RootBoost	PCA_based RootBoost
Colon	96.10 ± 0.59	95.48 ± 0.61^•^
CNS	95.00 ± 0.28	94.80 ± 0.59^•^
Leukaemia	98.77 ± 0.03	98.75 ± 0.31
Breast	97.88 ± 0.45	94.39 ± 0.49^•^
Lung	99.54 ± 0.11	98.11 ± 0.17^•^
Ovarian	99.40 ± 0.26	99.82 ± 0.08^o^
MLL	99.31 ± 0.55	98.86 ± 0.23^•^
SRBCT	99.59 ± 0.16	99.50 ± 0.31
Win tie loss		5/2/1

^•^Specifies that RotBoost is significantly better, and ^o^points out that RotBoost is notably worse at the significance level **α** = 0.05.

**Table 4:** Mean classification accuracy of each classification method against 8 different gene datasets.

Dataset	ICA-based RotBoost	Single Tree	Rotation Forest	AdaBoost	Bagging	SVMs
Colon	96.10 ± 0.59	93.80 ± 0.82^•^	95.21 ± 0.43^•^	94.97 ± 0.63^•^	94.92 ± 0.50^•^	96.13 ± 0.12
CNS	95.00 ± 0.28	89.92 ± 0.61^•^	92.37 ± 0.83^•^	95.09 ± 0.64	93.50 ± 0.79^•^	93.34 ± 0.10^•^
Leukemia	98.77 ± 0.03	96.60 ± 00.46^•^	97.97 ± 0.38^•^	98.22 ± 0.55^•^	97.47 ± 0.51^•^	95.64 ± 0.49^•^
Breast	97.88 ± 0.45	88.50 ± 0.72^•^	98.60 ± 0.63^o^	98.89 ± 0.47^o^	92.74 ± 0.45^•^	96.84 ± 0.02^•^
Lung	99.54 ± 0.11	94.36 ± 0.42^•^	97.56 ± 0.23^•^	96.30 ± 0.39^•^	97.08 ± 0.37^•^	95.56 ± 0.55^•^
Ovarian	99.40 ± 0.26	99.37 ± 0.12	99.77 ± 0.07^o^	99.57 ± 0.11	99.76 ± 0.08^o^	98.66 ± 0.35^•^
MLL	99.31 ± 0.55	96.03 ± 0.59^•^	97.61 ± 0.31^•^	97.63 ± 0.45^•^	97.11 ± 0.55^•^	96.80 ± 0.31^•^
SRBCT	99.59 ± 0.16	93.96 ± 0.59^•^	97.44 ± 0.41^•^	98.16 ± 0.39^•^	96.46 ± 0.58^•^	97.23 ± 0.44^•^
Win Tie Loss		7/1/0	6/0/2	5/2/1	7/0/1	7/1/0

^•^Specifies that RotBoost is significantly better, and ^o^points out that RotBoost is notably worse at the significance level **α** = 0.05.

**Table 5:** Kappa error diagram for Lung dataset (the centroids of ensembles).

Ensemble method	Kappa	Error
AdaBoost	0.22	0.30
Bagging	0.24	0.25
Rotation Forest	0.29	0.23
RotBoost (PCA)	0.58	0.09
RotBoost (ICA)	0.59	0.07
